# Hepatocellular Carcinoma: How the Gut Microbiota Contributes to Pathogenesis, Diagnosis, and Therapy

**DOI:** 10.3389/fmicb.2022.873160

**Published:** 2022-04-27

**Authors:** Wenyu Luo, Shiqi Guo, Yang Zhou, Jingwen Zhao, Mengyao Wang, Lixuan Sang, Bing Chang, Bingyuan Wang

**Affiliations:** ^1^Department of Gastroenterology, The First Affiliated Hospital of China Medical University, Shenyang, China; ^2^The Second Clinical College, China Medical University, Shenyang, China; ^3^Department of Gastroenterology, Shengjing Hospital of China Medical University, Shenyang, China; ^4^Department of Geriatric Medicine, The First Affiliated Hospital of China Medical University, Shenyang, China

**Keywords:** gut microbiota, gut-liver axis, hepatocellular carcinoma, diagnosis, treatment

## Abstract

The gut microbiota is gaining increasing attention, and the concept of the “gut-liver axis” is gradually being recognized. Leaky gut resulting from injury and/or inflammation can cause the translocation of flora to the liver. Microbiota-associated metabolites and components mediate the activation of a series of signalling pathways, thereby playing an important role in the development of hepatocellular carcinoma (HCC). For this reason, targeting the gut microbiota in the diagnosis, prevention, and treatment of HCC holds great promise. In this review, we summarize the gut microbiota and the mechanisms by which it mediates HCC development, and the characteristic alterations in the gut microbiota during HCC pathogenesis. Furthermore, we propose several strategies to target the gut microbiota for the prevention and treatment of HCC, including antibiotics, probiotics, faecal microbiota transplantation, and immunotherapy.

## Introduction

Liver cancer is a common malignancy. In 2020, primary liver cancer was the sixth most commonly diagnosed cancer (4.7% of total cancer cases) and the third leading cause of cancer death (8.3% of the total cancer deaths) worldwide, with approximately 906,000 new cases and 830,000 deaths (Sung et al., 2021). Primary liver cancer has high mortality, complex pathogenesis and drug resistance; in most regions, rates of both incidence and mortality are 2 to 3 times higher among men than among women (Villanueva, 2019; Sung et al., 2021). More than 80% of liver cancer cases occur in low- and middle-resource countries, particularly in East Asia and sub-Saharan Africa (Yang et al., 2019). Hepatocellular carcinoma (HCC) is the major histological subtype of liver cancer, accounting for 75–85% of all primary liver cancers, with a worldwide incidence of 850000 new cases per year (Forner et al., 2018; Sung et al., 2021). HCC is a typical outcome of a range of chronic liver diseases (CLDs), including alcoholic liver disease (ALD), virus-associated liver disease, non-alcoholic fatty liver disease (NAFLD) and cirrhosis due to other factors. It has been well established that the risk factors for HCC are liver fibrosis, hepatitis B virus (HBV)/hepatitis C virus (HCV) infection, heavy alcohol consumption, cholestasis, aflatoxin B1 ingestion, tobacco smoking, and NAFLD caused by obesity and/or insulin resistance (Forner et al., 2018; Kim and Viatour, 2020). To date, multiple hypotheses have been proposed to explain the evolutionary pattern of HCC (Craig et al., 2020). Currently, it is believed that five pathways and processes are associated with HCC progression, including the overexpression of telomerase, the activation of the Wnt/β-Catenin pathway, the inactivation of p53, the alteration of chromatin remodelling complexes with epigenetic regulators and constitutive activation of oxidative stress pathways (Forner et al., 2018). Recently, the relationship between the gut microbiota and HCC has gained increasing attention. The gut microbiota plays an important role in the physical state of the intestinal barrier as well as in the nutritional support of the liver, the maturation of the immune system, and the regulation of hepatocyte proliferation and differentiation (Zhou et al., 2020). There is evidence that the occurrence of HCC may be associated with microbiota dysregulation, abnormal bacterial metabolite production, and immune disorders between the host and the gut microbiota (Young, 2017). Through the anatomical connection of the gut-liver axis, leaky gut can enable multiple gut-microbiota-derived metabolites and gut-microbiota-associated molecular patterns to act on the hepatic immune system, ultimately affecting the occurrence and development of HCC. Treatments also vary at different stages of liver cancer. Despite the increasing popularity of some new therapies (targeted or biological therapies), their effects are still unsatisfactory (Zhou et al., 2020). Understanding the role of the gut microbiota in HCC may enable the development of more effective diagnostic and prevention approaches. Therefore, the treatment of HCC through adjustments targeting the gut microbiota will be a very promising research direction in the future. In this review, we provide insights into the role and related mechanisms of the gut microbiota in HCC development. We summarize recent findings on characteristic alterations in the gut microbiota in cirrhosis-HCC and strategies for targeting the gut microbiota to treat HCC.

## Physiological State of the Gut Microbiota

### Composition and Distribution of the Gut Microbiota

The intestine is the main organ for digestion and absorption and the largest immune organ of the human body, playing an important role in maintaining normal physiological function. The realization of intestinal function is accomplished with the assistance of various microorganisms settled in the intestine. The collection of various microorganisms, such as bacteria, archaea, eukarya, viruses and parasites, that colonize the intestine is termed the “gut microbiota” (Thursby and Juge, 2017). The number of microorganisms in the gut is vast, estimated to be approximately 10^13^ to 10^14^, with bacterial numbers being approximately 10 times the number of human cells; the number of genes contained within the gut microbiota is approximately 100 times the number in the genes in the human body (Gill et al., 2006; Acharya and Bajaj, 2017; Thursby and Juge, 2017). However, these estimations have been revisited in a recent study. After careful analysis and a more precise estimation, bacterial numbers were found to outnumber human cells by a ratio close to 1:1 (Sender et al., 2016). However, this article argued that updating the ratio of bacteria to human cells from 10:1 or 100:1 to closer to 1:1 does not take away from the biological importance of the microbiota (Sender et al., 2016). As a result, as an increasing number of researchers acknowledge, human-microbiome associations can be considered a superorganism (Yarza et al., 2008; Salvucci, 2019).

In recent years, our ability to survey the breadth of the gut microbiota has greatly improved due to the advent of culture-independent approaches such as high-throughput and low-cost sequencing methods (Thursby and Juge, 2017). By metagenomic analysis of DNA that codes for 16S rRNA, it is currently known that gut microorganisms basically belong to 7 phylogenetic gates, including *Firmicutes*, *Bacteroidetes*, *Proteobacteria*, *Actinobacteria*, *Verrucomicrobia*, *Fusobacteria* and *Cyanobacteria*, the first and second of which are the dominant bacteria (Yu and Morrison, 2004; Jandhyala et al., 2015). The majority of “good” bacteria within the human gut microbiota are represented by *Firmicutes* and *Cytophaga-Flavobacterium-Bacteroides* (Bibbò et al., 2016). By analysing comprehensive data from MetaHit and the Human Microbiome Project, the microbes in our bodies together make up 100 trillion cells and 2 kg of intestinal mass; we can identify 3.3 million effective reference genes for the human gut metagenome, approximately 150 times the number in the human genome (Li et al., 2014b; Hugon et al., 2015; Sánchez-Tapia et al., 2019). Cohort studies for Europeans found that the entire cohort harbours between 1000 and 1150 prevalent bacterial species, and each individual harbours at least 160 such species (Qin et al., 2010). However, the latest research identified 1,952 uncultured candidate bacterial species by reconstructing 92,143 metagenome-assembled genomes from 11,850 human gut microbiota (Almeida et al., 2019).

**FIGURE 1:**
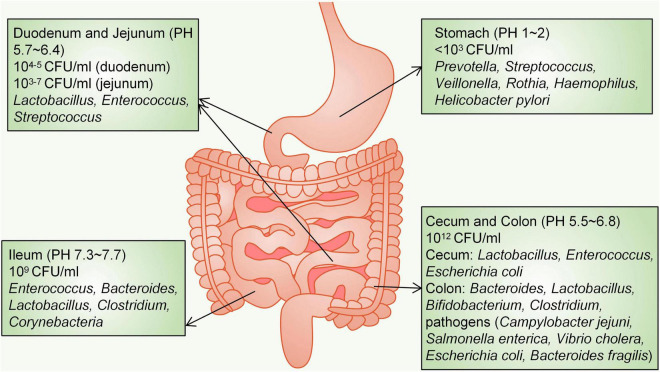
Distribution of microorganisms in the gastrointestinal tract. CFU, colony-forming units.

The distribution of different bacteria in the intestine varies. From the oesophagus distal to the rectum, there will be a marked difference in the diversity and number of bacteria, ranging from 10^1^ per gram of contents in the oesophagus to 10^12^ per gram of contents in the colon and distal gut (Jandhyala et al., 2015). More than 65% of the phylotypes in the stomach originate from the oral cavity, where five major phyla have been detected: *Firmicutes*, *Bacteroidetes*, *Actinobacteria*, *Fusobacteria* and *Proteobacteria* (Nardone and Compare, 2015). At the genus level, the healthy human stomach is dominated by *Prevotella*, *Streptococcus*, *Veillonella*, *Rothia*, *Haemophilus* and *Helicobacter pylori* (Hollister et al., 2014; Nardone and Compare, 2015). The acidic environment due to gastric juice (pH 1–2) limits the density of bacteria, which is less than 10^3^ colony-forming units (CFU)/ml (Adak and Khan, 2019). The small intestine is divided into three parts: the duodenum, jejunum and ileum. Bacterial diversity in the small intestine is lower than that in the colon (Yadav et al., 2018). A gradient of oxygen, antimicrobial peptides, bile acids and pH limits the bacterial density in the small intestine; therefore, the small intestine microbial community is dominated by fast-growing facultative anaerobes, mainly *Lactobacillaceae* and *Enterobacteriaceae*, to tolerate this environment (Donaldson et al., 2016). The duodenum is a hostile environment for microbial survival due to the addition of bile and digestive enzymes as well as the rapid transit of food, and the density (10^4–5^ CFU/ml) and diversity of bacteria are limited (Kastl et al., 2020). Three phyla (*Firmicutes*, *Proteobacteria*, and *Actinobacteria*) are dominant in the duodenum (Gong et al., 2019). The jejunum supports the diversity and density of bacterial colonization (10^3–7^ CFU/ml) and mainly supports the growth of Gram-positive aerobic and facultative anaerobic bacteria, including *Lactobacillus*, *Enterococcus* and *Streptococcus* (El Aidy et al., 2015; Adak and Khan, 2019). The acidity in the duodenum and jejunum is decreased significantly to approximately pH 5.7–6.4; however, in the ileum (pH 7.3–7.7), the density of bacteria is as high as 10^9^ CFU/ml, and ileal bacteria (aerobic bacteria dominate) include *Enterococcus*, *Bacteroides*, *Lactobacillus*, *Clostridium*, and *Corynebacteria* (Adak and Khan, 2019). In contrast, the distal part of the ileum close to the ileocecal valve is filled with anaerobes and colonic-like Gram-negative bacteria (Adak and Khan, 2019). The large intestine is mainly composed of the caecum and colon. Because of the slow rate of food transport and anaerobic conditions, the large intestine is the main place where undigested food absorbs water and ferments. In the large intestine (pH 5.5–6.8), the number of anaerobic bacteria is 100–1000 times higher than that of aerobic bacteria, and the bacterial density reaches 10^12^ CFU/ml (Eckburg et al., 2005). The predominant phyla that inhabit the large intestine include *Firmicutes* and *Bacteroidetes*, characterized by the presence of *Bacteroidaceae*, *Prevotellaceae*, *Rikenellaceae*, *Lachnospiraceae*, and *Ruminococcaceae* (Jandhyala et al., 2015; Donaldson et al., 2016). The caecal flora is mainly composed of *Lactobacillus*, *Enterococcus* and *Escherichia coli* (*E. coli*), and aerobic bacteria form the dominant species of the caecal microbial community (Donaldson et al., 2016). Abundant genera in the colon are *Bacteroides*, *Lactobacillus*, *Bifidobacterium*, and *Clostridium* (Yadav et al., 2018). The human colon also harbours primary pathogens, *e.g*., species such as *Campylobacter jejuni*, *Salmonella enterica*, *Vibrio cholera*, *Escherichia coli* (*E. coli*), and *Bacteroides fragilis*, but with a low abundance (0.1% or less of the entire gut microbiota) (Gillespie et al., 2011; Human Microbiome Project Consortium, 2012; Jandhyala et al., 2015; Figure 1).

### Effects of the Gut Microbiota on Liver Functioning

The gut microbiota can have a variety of physiological effects on the host, mainly by participating in metabolic processes and providing nutrition. The gut microbiota has very strong metabolic potential, and bile acids, short chain fatty acids, and others are important metabolites (Visconti et al., 2019). Bile acids (BAs) are main components of bile. Primary BAs mainly include cholic acid (CA) and chenodeoxycholic acid (CDCA), while secondary BAs mainly include deoxycholic acid (DCA) and lithocholic acid (LCA) (Di Ciaula et al., 2017). BAs can be combined with glycine (G-) or taurine (T-) to form conjugated-BAs. Primary BAs are synthesized by a series of enzymes in the liver, which are then discharged into the intestinal lumen through the gallbladder. The gut microbiota expresses bile salt hydrolase and 7-α dehydroxylase, which play a key role in the production of secondary BAs (Wahlström et al., 2016). Most BAs in the intestine return to the liver through the portal vein system, forming enterohepatic circulation. BAs can promote lipid absorption and control energy metabolism. At the same time, bile acids mediate a variety of downstream signal pathways and widely participate in the process of immune regulation. Therefore, due to the complexity of the gut microbiota itself, studying its metabolites may provide additional important valuable information for pathogenesis and treatment.

The gut and liver communicate *via* tight bidirectional links through the biliary tract, portal vein and systemic circulation, forming the gut-liver axis (Tripathi et al., 2018). The normal gut microbiota can interact with the host’s immune system to form the gut barrier, thereby preventing pathogen and toxin invasion and protecting the body from disease (Sidhu and van der Poorten, 2017). However, microbial dysbiosis can cause the disturbance of gut barrier function, and a variety of microbial associated harmful factors enter the liver along the gut-liver axis, ultimately leading to the occurrence of HCC.

## Mechanisms by Which the Gut Microbiota Mediates the Development of Hepatocellular Carcinoma

### Bile Acids

During the development of HCC, the accumulation of hydrophobic BAs, including CA, G-CA, CDCA, DCA, LCA, induce a variety of signals that prompt hepatocyte death (Thomas et al., 2008). Bile salt hydrolase is a key enzyme that converts conjugated-BAs into more hydrophobic free-BAs, while studies have found abundant bile salt hydrolase expression in *Bacteroides*, *Lactobacillus*, and *Bifidobacterium* (Swann et al., 2011). The TNF-related apoptosis inducing ligand receptor (TRAILR) and Fas death receptor signalling pathways are two important downstream pathways, and bile-acid-mediated hepatocyte apoptosis not only activates ligand-independent death receptor oligomerization but also regulates the sensitivity of death-receptor-related signalling pathways (Wei et al., 2020). BAs stimulate cFLIP (cellular FLICE-like inhibitory protein) phosphorylation, which in turn promotes the activation of caspases 8 and 10 (Higuchi et al., 2003). Activated caspases 8 and 10 cleave bid into tBid and enter it mitochondria with Bax (both Bax and tBid are proapoptotic BcL-2 family proteins) (Wei et al., 2020). Hepatocytes lacking mitochondrial DNA (mtDNA) are fairly resistant to bile-acid-induced apoptosis, and mitochondria have therefore been suggested to be a likely involved central component. In the human hepatoma SK-Hep-1 cell subline with complete mtDNA depletion, bile-acid-induced proapoptotic signalling was reduced, as indicated by decreased expression rates of Bax-α/B cell lymphoma-2 (Bcl-2), Bcl XS/Bcl-2, and Bcl XS/Bcl XL, as well as reduced bile-acid-induced reactive oxygen species (ROS) production (Marin et al., 2013). Mitochondria may also release damage-associated molecular patterns after injury, which in turn activate Toll-like receptors (TLR) 9, stimulating the expression of inflammatory cytokines (Li et al., 2017). A large number of mitochondria within hepatocytes release excessive ROS and cytochrome C upon stimulation by BAs, activating the caspase cascade and inducing apoptosis through caspase-3-related signalling pathways (Paradies et al., 2009). Hydrophobic BAs (DCA and G-CDCA) release Ca^2+^ to the cytoplasm by inducing endoplasmic reticulum (ER) stress, Ca2+ and ROS promote each other in the process of oxidative stress, forming a vicious cycle (Wei et al., 2020). Activated Ca2+ can also promote the expression of Bax through positive feedback amplification of the calcium/calmodulin-dependent protein kinase II (CaMKII)-C/EBP homologous protein (CHOP) signalling pathway (Li et al., 2009; Santos et al., 2009; Wei et al., 2020).

Bile acids promote the release of inflammatory cytokines through multiple pathways. Toxic BAs (such as T-LCA) can directly damage the plasma membrane and activate protein kinase C (PKC), which in turn activates the p38 MAPK (mitogen-activated protein kinase) pathway, leading to the activation of downstream p53 and NF-κB (Anwer, 2012; Jia et al., 2018). Activated NF-κB translocates to the nucleus, where it promotes the transcription of the genes encoding TNF-α, IL-1β, and IL-6, thereby promoting a continuous cycle of inflammation (Luedde and Schwabe, 2011; Jia et al., 2018). IL-6 also activates the Janus kinase (JAK) signal transducer and activator of the STAT3 (signal transducer and activator of transcription 3) pathway, leading to adverse effects in tumours, such as reduced apoptosis in HCC cells (Huang et al., 2015; Jung et al., 2015). DCA and other hydrophobic BAs-induced perturbations of the cell membrane also activate cytosolic phospholipase A2 (PLA2), which in turn releases arachidonic acid (AA) through cyclooxygenase (COX) and lipoxygenase (LOX), ultimately leading to an increase in ROS content (Bernstein et al., 2005; Jia et al., 2018). ROS also directly activate NF-κB (Ouyang et al., 2013). CDCA and DCA can upregulate the expression of early growth response gene 1 (Egr-1) by activating epidermal growth factor receptor (EGFR), which leads to the production of proinflammatory factors such as vascular endothelial cell adhesion molecule 1 (vcm-1) and IL-1β by hepatocytes (Yan et al., 2000; Wei et al., 2020).

Farnesoid X receptor (FXR) is the most important nuclear receptor for BAs to mediate the process of HCC development. The order of binding potency of BAs to FXR is CDCA > LCA = DCA > CA (Schubert et al., 2017). Overall, FXR inhibits hepatocarcinogenesis mainly through the following mechanisms: (1) FXR maintains normal liver homeostasis and the metabolism of BAs, glucose, and lipids; (2) FXR promotes hepatocyte survival; (3) FXR suppresses liver inflammation while facilitating regeneration and repair after liver injury; and (4) FXR increases the expression of partial tumour suppressor genes, inhibiting the transcription of multiple oncogenes (Huang et al., 2015). A downregulation of FXR expression in hepatocytes is closely associated with the development of HCC. In samples of stage I HCC, the expression level of FXR has been shown to be decreased to 40% of normal and further decreased at a later stage of the disease (Wolfe et al., 2011). This study also observed that spontaneous hepatocarcinogenesis in FXR knockout mice was associated with sustained activation of the Wnt/β-catenin pathway (Wolfe et al., 2011). Under pathological conditions of cholestasis, toxic BAs such as (T-DCA)-, T-CA and DCA reduce the expression of sirtuin 1 (SIRT1, a transcriptional regulator of FXR) in hepatocytes (Zhao et al., 2019). However, this contradicts previous experimental findings that the overexpression of SIRT1 in transgenic mice leads to decreased FXR expression and ultimately causes HCC (García-Rodríguez et al., 2014). During liver injury, high levels of TNF-α, IL-1β, IL-6, and Fas ligand (FasL) were shown to rapidly decrease the expression level of FXR before the activation of apoptotic signalling, and the underlying mechanism was that FXR expression was reduced by inhibiting the activity of hepatocyte nuclear factor 1α (HNF 1α) on the FXR gene promoter (Huang et al., 2015; Wei et al., 2020). TNF-α-induced activation of hepatic NF-κB in mice suppresses hepatic FXR expression, and NF-κB p50/p65 dimers can also directly bind to the FXR promoter and suppress its transcription (Gadaleta et al., 2011; Jia et al., 2018). Takeda G protein-coupled receptor 5 (TGR5), another important receptor for BAs, is thought to play a role as well. The methylation of the TGR5 promoter is significantly more frequent in HCC than in chronic hepatitis B, and the hypermethylated TGR5 serum cell-free DNA promoter may serve as a biomarker for HCC surveillance (Han et al., 2014).

**FIGURE 2:**
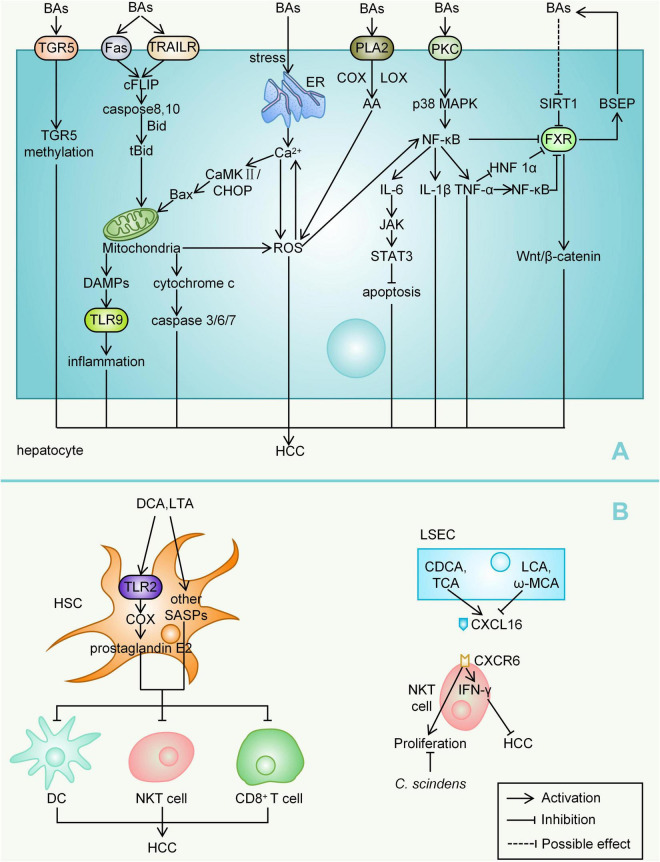
Mechanisms by which BAs mediate HCC. **(A)** Bile acids, as important metabolites of the gut microbiota, can cause inflammatory responses, cell death, ROS accumulation, reduction of apoptosis, and so on, mainly by mediating complex signalling pathways within hepatocytes, ultimately leading to the development of HCC. **(B)** BAs can also act on other liver cells such as HSC, LSEC, NKT cell, and so on, thereby affecting HCC progression. BAs, bile acids; TRAILR, TNF-related apoptosis inducing ligand receptor; cFLIP, Cellular FLICE-like inhibitory protein; mtDNA, mitochondrial DNA; Bcl-2, B cell lymphoma-2; ROS, reactive oxygen species; TLR, Toll-like receptor; ER, endoplasmic reticulum; CaMKII, calcium/calmodulin-dependent protein kinase II; CHOP, C/EBP homologous protein; PKC, protein kinase C; MAPK, mitogen-activated protein kinase; JAK, Janus kinase; STAT3, signal transducer and activator of transcription 3; PLA2, phospholipase A2; AA, arachidonic acid; COX, cyclooxygenase; LOX, lipoxygenase; CDCA, chenodeoxycholic acid; DCA, deoxycholic acid; Egr-1, early growth response gene 1; EGFR, epidermal growth factor receptor; vcm-1, vascular endothelial cell adhesion molecule 1; FXR, Farnesoid X receptor; TDCA, taurodeoxycholic acid; TCA, taurocholic acid; SIRT1, sirtuin 1; FasL, fas ligand; HNF 1α, hepatocyte nuclear factor 1α; TGR5, Takeda G protein-coupled receptor 5; HSC, hepatic stellate cell; LTA, lipoteichoic acid; SASP, senescence-associated secretory phenotype; DCs, dendritic cells; NKT cell, natural killer T cell; CXCR, C-X-C chemokine receptor type; LSEC, liver sinusoidal endothelial cell; LCA, lithocholic acid; ω-MCA, ω-muricholic acid; NK cell, natural killer cell.

Deoxycholic acid plays an important role in promoting the progression of HCC, and its production depends on the 7α-dehydroxylation of primary BAs by intestinal bacteria, especially *Clostridium* clusters. In the non-alcoholic steatohepatitis-HCC mouse model induced by high-fat diet, Gram-positive bacterial strains increased significantly, especially specific *Clostridium* clusters (Yoshimoto et al., 2013). For hepatic stellate cells (HSCs), DCA and lipoteichoic acid (LTA) synergistically induce senescence-associated secretory phenotypes (SASPs), including IL-6, growth-regulated oncogene-α, chemokine 9 and prostaglandin E2, resulting in a tumorigenic microenvironment that promotes HCC development in mice (Yoshimoto et al., 2013; Jia and Jeon, 2019). Prostaglandin E2 is key for tumour development. LTA and DCA synergistically enhance TLR2-mediated signalling and induce the overexpression of COX-2, which catalyses the rate-limiting step of prostaglandin (Schroeder and Bäckhed, 2016; Jia and Jeon, 2019). COX-2 and prostaglandin cascades promote tumour development and immune escape by suppressing dendritic cells (DCs), natural killer T (NKT) cells, and CD8^+^ T cells (Liu B. et al., 2015; Loo et al., 2017). In the liver, BAs mediate liver tissue damage mainly through neutrophils and Kupffer cells (Li et al., 2017). A 2018 study targeted BAs with NKT cells. NKT cells express C-X-C chemokine receptor type (CXCR) 6 on their surface, and the expression of the CXCR6 ligand, CXCL16, on liver sinusoidal endothelial cells (LSECs) regulates NKT cell accumulation (Geissmann et al., 2005; Ma et al., 2018). CXCR6 accumulation in hepatic NKT cells increases IFN-γ production and promotes tumour growth inhibition. The primary BAs CDCA and T-CA increased CXCL16 expression, whereas the secondary BAs LCA or ω-muricholic acid (ω-MCA) did the opposite (Ma et al., 2018). Studies have confirmed that *C. scindens* rapidly reduces the number of hepatic NKT cells (Ma et al., 2018) (Figure 2). It is important to note that the immune system and the composition of the gut microbiota differ between mice and humans, with approximately 30% of hepatic lymphocytes in mice being NKT cells compared with only approximately 1% in humans (Schramm, 2018). Mice and humans also differ substantially in their bile acid composition. Therefore, caution must be taken when translating findings from mice to humans.

### Effects of Toll-Like Receptors 4

Among various TLRs, TLR4 is considered to be the most important receptor to mediate HCC. TLR4 can be expressed by various liver cells, including hepatocytes, Kupffer cells, HSCs, biliary epithelial cells, LSECs, DCs, natural killer (NK) cells, B cells, and T cells (Sun et al., 2016). TLR4 primarily recognizes lipopolysaccharide (LPS),in addition, TLR4 also binds free fatty acids and high-mobility group box 1 (HMGB1) (Schwabe et al., 2006; Sun et al., 2016).

Lipopolysaccharide is a component of the cell wall of Gram-negative bacteria. It was found that high-fat diet could increase the level of LPS in the blood of mice, accompanied by the reduction in abundance of *Lactobacillus* and *Bifidobacterium* (Cani et al., 2008). DCA and CDCA can also increase intestinal permeability by regulating the tight junctions between the intestinal epithelium, and a large amount of LPS enters the blood circulation through the damaged intestinal barrier (Raimondi et al., 2008). LPS-TLR4 signalling promotes cell survival and proliferation in HCC (Wang L. et al., 2013). Mice deficient in TLR4 have less liver injury and inflammatory responses than wild-type mice, indicating that LPS from the gut microbiota is the major source responsible for inflammatory signals (Weiss, 2012). The microbiota can promote hepatocarcinogenesis by activating TLR4 *via* diethylnitrosamine and hepatotoxin carbon tetrachloride (Dapito et al., 2012). TLR4 expression in HCC tissues (77.8% positive rate) was significantly higher than that in adjacent noncancerous tissues (20%), whereas genetic TLR4 inactivation, gut sterilization or germ-free status could decrease HCC development by approximately 80% (Dapito et al., 2012; Kang et al., 2018).

Toll-like receptors 4 mediates multiple signalling pathways. Findings from studies in chimeric mice suggest that the LPS-TLR4 pathway promotes HCC mainly by increasing proliferative and antiapoptotic signals in resident hepatocytes of non-myeloid origin (Dapito et al., 2012). MAP kinase 4 (MKK4)/c-Jun N-terminal kinase (JNK) signalling, which can be activated by LPS treatment, significantly enhanced the production of matrix metallopeptidase 2 (MMP2), MMP9, IL-6, and TNF-α and the invasiveness of HCC cells (Dong et al., 2015). LPS can directly activate JNK/MAPK signalling in HCC cells *via* TLR4 to induce the invasive capacity and epithelial mesenchymal transition (EMT) of HCC cells (Li et al., 2014a). Studies in a nude mouse model showed that blocking NF-κB could significantly inhibit the expression of the transcription factor Snail, thus inhibiting the occurrence of EMT (Jing et al., 2014). TLR4/CXCL9/PREX-2 (phosphatidylinositol-3, 4, 5-trisphosphate RAC exchanger 2) is an important pathway in the pathogenesis of liver cirrhosis and can be activated by thioacetamide (Elshaer et al., 2019). However, this pathway can be significantly inhibited upon the addition of *Lactobacillus plantarum* in rats (Elshaer et al., 2019). The transcription factor STAT3 (which can be activated by IL-6) can regulate a series of genes, including cyclin D1, Bcl-2, c-myc and IL-10, that promote cell growth and angiogenesis (Lin et al., 2016). One study, which evaluated the role of STAT3 in a xenograft model in nude mice, reported a novel mechanism; namely, STAT3 activated by LPS increases the production of vascular endothelial growth factor (VEGF) by HCC cells, thereby promoting the proliferation of tumours while stimulating the migration of endothelial cells and angiogenesis in HCC (Wang et al., 2019). Inhibition by geniposide, an antitumour agent, led to the shutdown of the TLR4 pathway and a decline in STAT3/specificity protein 1 (SP1)-dependent VEGF production (Zhang et al., 2020). However, the addition of LPS restored STAT3/SP1-associated VEGF production for HCC angiogenesis (Zhang et al., 2020). COX-2, one of the cytokines associated with tumorigenesis, can be coregulated by NF-κB and STAT3, and COX-2 dysregulation leads to an increase in its main metabolite prostaglandin E2 (Lin et al., 2016). Studies have identified a COX-2/prostaglandin E2/STAT3 positive feedback loop in HCC cells, and the activation of TLR4 can cause the activation of this loop (Lin et al., 2016). The tumour suppressor microRNA-122 (miR-122) is thought to decrease the expression of VEGF, IL-6, COX-2, prostaglandin E2 and MMP-9 (Wei et al., 2019). The downregulation of miR-122 promotes immune escape of HCC by targeting TLR4 and the related phosphoinositide 3-kinase (PI3K)/Akt/NF-κB signalling pathway (Wei et al., 2019).

**FIGURE 3:**
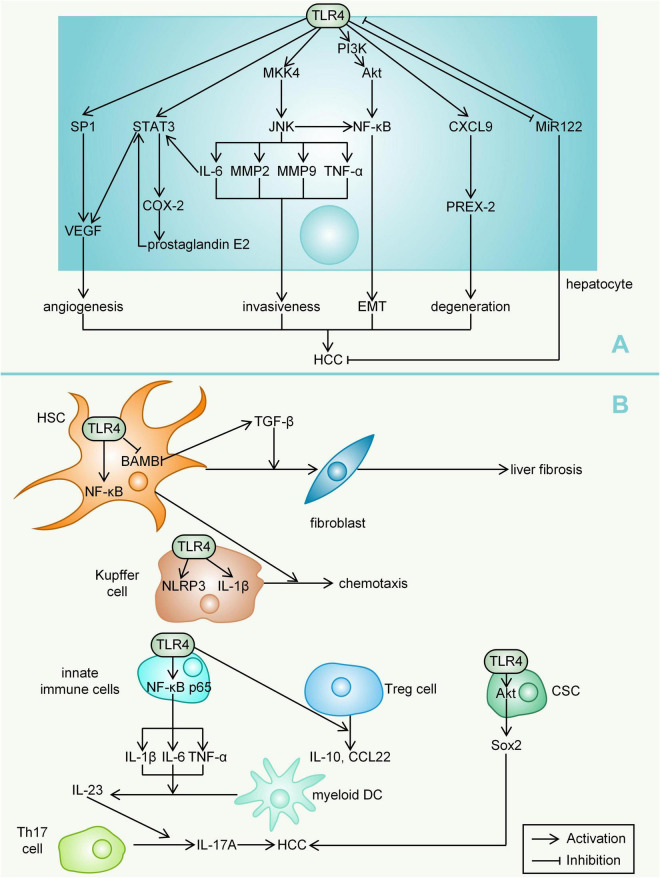
Mechanisms by which TLR4 mediates HCC. **(A)** For hepatocytes, TLR4 can lead to angiogenesis (activation of VEGF), invasiveness (through multiple inflammatory mediators), EMT, degeneration (activation of PREX-2) and downregulation of miR122, ultimately promoting the development of HCC. **(B)** In other liver cells, TLR4 expression also activates signals (such as NF-κB, interleukin and TNF-α) that promote HCC development. LPS, lipopolysaccharide; HMGB1, high-mobility group box 1; MKK4, MAP kinase 4; JNK, c-Jun N-terminal kinase; MMP, matrix metallopeptidase; EMT, epithelial mesenchymal transition; PREX-2, phosphatidylinositol-3, 4, 5-trisphosphate RAC exchanger 2; VEGF, vascular endothelial growth factor; SP1, specificity protein 1; miR-122, microRNA-122; PI3K, phosphoinositide 3-kinase; Akt, protein kinase B; BAMBI, bone morphogenetic protein and activin membrane bound inhibitor; TGF, transforming growth factor; NLRP3, NLR family pyrin domain-containing 3; Th17 cell, T helper 17 cell; Pten, phosphatase and tensin homologue; Treg cell, regulatory T cell; CCL22, C-C class chemokine 22; SOX2, SRY-box containing gene 2; CSC, cancer stem cell.

The promotion of early TLR4-dependent HCC is mainly mediated by the secretion of TLR4-dependent growth factors (such as epiregulin) by HSCs (Dapito et al., 2012). LPS, which promotes the transformation of HSCs into fibroblasts through the TLR4-NF-κB signalling pathway, induces liver fibrosis (Seki et al., 2007; Qi et al., 2020). Quiescent HSCs highly express BAMBI (bone morphogenetic protein and activin membrane bound inhibitor), but their transcriptional activity is inhibited after LPS treatment, which contributes to TLR4-mediated enhancement of transforming growth factor (TGF)-β signalling in HSCs during liver fibrosis (Liu et al., 2014). Chemokines secreted by HSCs upon LPS stimulation can promote Kupffer cell chemotaxis (Yang et al., 2015). Kupffer cells express significantly more NLR family pyrin domain-containing 3 (NLRP3) and IL-1β after LPS stimulation (Yu et al., 2017). LPS-induced hepatic innate immune responses in mice are regulated by NF-κB p65 (Zarate et al., 2020). LPS-mediated activation of TLR4 can lead to the degradation of the inhibitory proteins (inhibitor of κBα and inhibitor of κBβ), allowing nuclear translocation of NF-κB subunits and finally resulting in the upregulation of key proinflammatory genes (Christian et al., 2016; Zarate et al., 2020). The activation of NF-κB promotes the production of proinflammatory cytokines, such as IL-1β, IL-6, and TNF-α. These cytokines stimulate myeloid DCs to secrete IL-23, which promotes the differentiation, proliferation, and maintenance of T helper 17 (Th17) cells (Blauvelt et al., 2015; Kang et al., 2018). LPS can promote the mRNA transcription and protein expression of IL-23 and IL-17A; that is, LPS participates in the proliferation and metastasis of HCC cells by regulating the IL-23/IL-17A axis (Kang et al., 2018). High doses of LPS (such as 1 μg/ml) provide both priming and activation signals in human monocytes and macrophages, leading to mature IL-1β secretion (Wang H. et al., 2013; Yu et al., 2017). One study analysed the role of macrophages in steatohepatitis-related HCC of hepatocyte-specific Pten (phosphatase and tensin homologue)-deficient mice. TLR4 deficiency, but not TLR2 deficiency, in hepatocyte-specific Pten-deficient mice suppressed tumour growth and hepatic inflammation (Miura et al., 2016). The clustering of regulatory T (Treg) cells in hepatoma cell lines could be indirectly promoted by the interaction of TLR4 with macrophages, accompanied by the upregulation of IL-10 and C-C class chemokine 22 (CCL22) expression (Yang et al., 2012). In addition, LPS increased Sox2 (SRY-box containing gene 2, a stemness marker gene) expression *via* the TLR4-Akt pathway, thereby upregulating the cancer stem cells (CSCs) of HCC and increasing the risk of recurrence after liver transplantation (Zhou et al., 2019). There is an association between TLR4 expression and CSC characteristics, and TLR4 may act as a CSC marker, promoting tumour invasion and migration (Liu W.T. et al., 2015; Figure 3).

### Other Mechanisms

#### Other Metabolites

A high cholesterol diet was found to induce NAFLD-HCC formation, which was accompanied by gut microbial dysbiosis (*Bifidobacterium and Bacteroides* depleted) and altered flora metabolites (serum taurocholic acid was increased while 3-indolepropionic acid was depleted) in patients (Zhang et al., 2021). A recent study found a link between short chain fatty acids (SCFAs) and HCC development. HBx, an HBV-encoded oncoprotein, contributes to the pathogenesis of HCC. Feeding HBx transgenic mice with SCFAs promoted the expression of the tumour suppressor disabled homologue 2 and downregulated multiple inflammatory signalling pathways, thereby delaying HCC development (McBrearty et al., 2021). At present, we have only a somewhat clear understanding of the mechanism of BAs-mediated HCC, although many metabolites have been found to play a role in tumour progression and metastasis. It will be an attractive prospect to study the metabolites or bacterial components that can regulate the immune response to verify whether they can play a role in promoting the development of HCC.

#### Toxins

It has been suggested that bacterial toxins in the gut microbiota can indirectly damage DNA through ROS, or directly damage host DNA. Cancer occurs when DNA damage exceeds the repair capacity of the host cell (Garrett, 2015). Aflatoxin is a well-known powerful carcinogen of HCC. At present, a variety of mycotoxins, including aflatoxin, have been found to affect the composition of the gut microbiota (Liew and Mohd-Redzwan, 2018). If the specific gut microbiota-mycotoxin-HCC process can be clarified, it will make an innovative contribution to knowledge regarding the pathogenesis of HCC. Toxic trace elements can also mediate HCC through the disturbance of intestinal flora. A study proved that arsenic intake was able to create an increased number of Gram-negative bacteria and elevated intestinal permeability, which in turn translocated large amounts of LPS to the liver and eventually caused the occurrence of HCC (Choiniere and Wang, 2016). In conclusion, further studies are needed on the mechanisms through which the gut microbiota mediates HCC to determine whether there are other metabolites or other pathways through which the gut microbiota might exert carcinogenic effects.

## Links Between Cirrhosis-Hepatocellular Carcinoma and Changes in the Gut Microbiota

The earliest formal definition of cirrhosis was “a chronic, progressive, destructive lesion of the liver combined with reparative activity and contact on the part of the connecting tissue” (Barnett, 2018). Almost all HCC occurs in patients with pre-existing advanced liver fibrosis and cirrhosis, and approximately 70%-90% of patients with HCC have concomitant cirrhosis (Llovet et al., 2016; Roehlen et al., 2020). In fact, cirrhosis due to any cause increases the risk of HCC (Lok et al., 2009; Villanueva, 2019). In recent years, much attention has been given to the correlation between the development of cirrhosis and a dysregulated gut microbiota, which is similar to a metaphor made by an article, namely, the relationship of the “chicken” and the “egg” (Arab et al., 2018). Gut microbiota dysregulation in cirrhosis may be related to diet, antibiotic abuse, alterations in BAs and intestinal motility, changes in gastric pH, and mucosal immune function impairment (Arroyo et al., 2016). Bacteria and their metabolites enter the lymph node pathway and blood circulation through a leaky gut, increasing portal vein pressure and promoting irreversible fibrosis of the liver (Sorribas et al., 2019). In the development of liver cirrhosis, portal pressure increases significantly, leading to bleeding, ascites and infection. These conditions will further damage microcirculation and increase the permeability of the gut barrier (Sorribas et al., 2019; Trebicka et al., 2021).

In fact, the structure and function of the gut microbiota in patients with liver cirrhosis and HCC are very different from those in healthy people. A study constructed the gene catalogue of 98 Chinese patients with liver cirrhosis and 83 healthy controls and found that as many as 75245 genes were very different between patients and healthy people (Qin et al., 2014). *Veillonella*, *Streptococcus*, *Clostridium* and *Prevotella* were enriched in the cirrhosis group, while *Eubacterium*, *Alistipes* and *Faecalibacterium prausnitzii* were more abundant in healthy people (Garcia-Tsao and Wiest, 2004; Qin et al., 2014). Interestingly, a high proportion of oral bacteria, such as *Streptococcus* and *Veillonella*, were found in patients with liver cirrhosis, suggesting that oral microorganisms had invaded the intestinal tract and contributed to liver cirrhosis (Qin et al., 2014; Bajaj et al., 2015a; Sanduzzi Zamparelli et al., 2017). Another earlier study found that the proportion of *Bacteroidetes* was significantly decreased in a cirrhosis group, while *Proteobacteria* and *Fusobacteria* were highly enriched (Chen et al., 2011). Researchers have identified the key systemic types of response to cirrhosis, most of which are *Lachnospiraceae*, *Streptococcaceae* and *Veillonellaceae* (Chen et al., 2011). In addition, *Candida* and other fungi were also found in patients with cirrhosis (Bajaj et al., 2018b). There are also differences in the gut microbiota between compensated and decompensated cirrhosis. *Proteobacteria* levels, especially *Enterobacteriaceae*, were significantly higher in patients with decompensated cirrhosis than in those with nondecompensated cirrhosis (Bajaj et al., 2015b). There were also other significant changes in the decompensated period, including *Haemophilus parainfluenzae*, *Veillonella parvula*, *Veillonella dispar*, *Veillonella sp.*, *Veillonella atypica*, *Fusobacterium nucleatum*, *Campylobacter sp.*, *Aggregatibacter segnis*, *Streptococcus vestibularis*, and *Streptococcus oralis* (Bajaj et al., 2015b). In addition, the species *Staphylococcus* and *Enterococcus* were increased, while some beneficial commensals, such as *Lachnospiraceae*, *Ruminococcus*, and *Clostridium XIVa*, were decreased (Albillos et al., 2020).

During the cirrhosis-HCC transition phase, the microbiota undergoes several additional alterations. The phylum *Actinobacteria* was shown to be increased in early HCC vs. cirrhosis (Ren et al., 2019). Correspondingly, *Gemmiger* and *Parabacteroides* were enriched (Ren et al., 2019). HCC-cirrhotic patients were also found to have a significant overrepresentation of *Clostridium* and *CF231* and reduced *α-Proteobacteria* abundance compared to cirrhotic patients without HCC (Lapidot et al., 2020). In addition, a unique microbial signature of *Akkermansia muciniphila* was observed in HCC-cirrhotic patients (Lapidot et al., 2020). In one study, 300 primary liver cancer patients were selected and divided into an early-stage liver cancer group (higher abundance of *Clostridiales*, *Firmicutes* and *Streptococcus*), an intermediate-stage liver cancer group (higher abundance of *Ruminococcaceae*, *Pasteurellaceae*, *Tanticharoenia*, and *Vagococcus*), and an advanced-stage liver cancer group (higher abundance of *Bifidobacteriales*, *Actinobacteria*, *Barnesiella*, *Porphyromonadaceae*, and *Pseudomonadales*) (Jiang et al., 2020). As HCC was aggravated, plasma endotoxin and VEGF levels were also significantly elevated (Jiang et al., 2020). As suggested by the findings from another study, interactions between the gut microbiota and microRNAs play a key role in vascular dysfunction and HCC (Feng et al., 2018). Some specific microorganisms may serve as markers for the diagnosis of HCC. A study in liver-specific tuberous sclerosis complex 1 knockout mice found that a decrease in the relative abundance of anaerobes and an increase in the relative abundance of facultative anaerobes could serve as risk indicators for liver cancer in females (Huang et al., 2018). *Parapretella*, *Parapretellaceae*, and *Prevotella* could be used as risk indicators for liver cancer in males (Huang et al., 2018). In another study, a more comprehensive index, termed the degree of dysbiosis (*D*_*dys*_), was introduced. The investigators found that the *D*_*dys*_ of patients with primary liver cancer was significantly different from that of healthy controls (*P* >0.01) (Ni et al., 2019). Many genera of *Proteobacteria*, such as *Enterobacter* and *Haemophilus*, were also increased in faecal samples from patients with primary liver cancer (Ni et al., 2019). Changes in the gut microbiota in cirrhosis and HCC may serve as a basis for diagnosis and screening. It is worth noting that the samples and sample sizes selected in these different studies varied, and different patients may have some differences in the distribution of the gut microbiota due to differences in sex, ethnicity, dietary habits and other factors (Table 1).

**TABLE 1:** Current findings of gut microbial characteristics for cirrhosis-hepatocellular carcinoma patients.

Phylum	Family	Genus	Species
*Firmicutes*	*Staphylococcaceae*	*Staphylococcus↑*	
	*Enterococcaceae↑*	*Vagococcus↑*	
		*Enterococcus↑*	*Enterococcus faecalis↑*
	*Lactobacillaceae*	*Lactobacillus↓*	
	*Streptococcaceae↑*	*Streptococcus↑*	*Streptococcus anginosus↑, Streptococcus vestibularis↑, Streptococcus oralis↑*
	*Clostridiaceae*	*Clostridium↑*	*Clostridium perfringens↑, Clostridium cluster XI↑, Clostridium XIVa↑*
	*Lachnospiraceae↓*	*Coprococcus↓*	*Coprococcus comes↓, Coprococcus spp.↓*
		*Blautia↓, Dorea↑, Anaerostipes↓*	
	*Ruminococcaceae↓*	*Ruminococcus↓*	*Faecalibacterium prausnitzii↓*
		*Eubacterium↓*	
	*Oscillospiraceae↓*		
	*Veillonellaceae↑*	*Veillonella↑*	*Veillonella atypica↑, Veillonella dispar↑, Veillonella sp.↑, Veillonella parvula↑*
*Bacteroidetes*	*Bacteroidaceae↑/↓*	*Bacteroides↓*	
	*Porphyromonadaceae↓*		
	*Prevotellaceae↑/↓*	*Prevotella↑/↓*	
*Proteobacteria↑*	*Enterobacteriaceae ↑*	*Escherichia↑, Klebsiella↑, Enterobacter↑*	
	*Pseudomonadaceae↑*	*pseudomonadales↑*	
	*Campylobacteraceae*	*Campylobacter*	*Campylobacter sp.↑*
	*Pasteurellaceae↑*		*Haemophilus parainfluenzae↑, Aggregatibacter segnis↑*
	*Desulfobacteraceae*	*Desulfococcus↑*	
*Actinobacteria*	*Atopobiaceae↑*		
	*Bifidobacteriaceae↓*	*Bifidobacterium↓*	
*Fusobacteria*	*Fusobacteriaceae↑*		*Fusobacterium nucleatum↑, Cetobacterium↓*
*Verrucomicrobia*		*Akkermansia muciniphila↓*	

*↑/↓ indicated that different studies obtained conflicting results (Chen et al., 2011; Bajaj et al., 2014, 2015b; Qin et al., 2014; Arab et al., 2018; Ni et al., 2019; Ren et al., 2019; Albillos et al., 2020; Huang et al., 2020; Jiang et al., 2020; Lapidot et al., 2020; Trebicka et al., 2021).*

The essence of the gut microbial changes was that beneficial indigenous bacteria such as *Lactobacillus*, *Bifidobacterium*, and *Bacteroides* species decreased, while potentially pathogenic bacteria such as *Proteobacteria* (particularly *Enterobacteriaceae*), *Fusobacterium spp.*, *Veillonellaceae* and *Streptococcaceae* increased (Arab et al., 2018). Changes in the relative content of bacteria influenced the metabolic status of patients with cirrhosis-HCC to a greater extent. There are two controlled experiments that demonstrate a decrease in butyrate-producing genera and an increase in LPS-producing genera of the gut microbiota during the course of cirrhosis and early HCC (Qin et al., 2014; Ren et al., 2019; Zheng et al., 2020). The first comprehensive shotgun sequence analysis in Chinese patients with cirrhosis highlighted the depletion of *F. prausnitzii* from *Ruminococcaceae* and of *Coprococcus spp.* from *Lachnospiraceae* (Qin et al., 2014; Trebicka et al., 2021). This suggests a protective effect of butyrate and a damaging effect of LPS. A comparison of the microbial metabolites in cirrhotic patients with those in healthy individuals revealed that seven metabolic pathways were significantly enhanced in the former, and the metabolism of specific amino acids and sugars was carried out at high levels (Wei et al., 2018). Functional analysis of 182 faecal samples from patients with cirrhosis revealed an enrichment of pathways involved in ethanol production, γ-aminobutyric acid metabolism, and endotoxin biosynthesis (Solé et al., 2021). BAs are key factors linking the gut microbiota and HCC. In one study, stool samples were collected from HBV-associated HCC patients and healthy volunteers for 16S rRNA sequencing, and it was found that *Bacteroides*, *Lachnospiracea incertae sedis*, and *Clostridium XIVa* were enriched in HCC patients with higher tumour burden (Huang et al., 2020). BAs produced by their metabolism caused changes in the tumour immune microenvironment (Murakami et al., 2018; Huang et al., 2020). *Clostridium XIVa* functions in BA 7α-dehydroxylation and influences BA-controlled NKT cell accumulation (Ma et al., 2018). Indeed, in end-stage liver disease, the capacity of the liver to synthesize BAs is reduced, whereas the proportion of secondary BAs in the gut is relatively elevated. These factors all disrupt the gut barrier, with concomitant attenuation of FXR signalling (Arab et al., 2018; Albillos et al., 2020).

## Targeting the Gut Microbiota for the Treatment of Hepatocellular Carcinoma

### Antibiotics

Antibiotics can inhibit the proliferation and translocation of intestinal flora by inhibiting bacterial DNA transcription, protein synthesis and other biological processes to reduce the liver’s acceptance of proinflammatory signals from the leaking gut. Rifaximin is a semisynthetic, water-insoluble, non-systemic antibiotic with low gastrointestinal absorption (Scarpignato and Pelosini, 2005). At present, the only acceptable indication for rifaximin in patients with liver cirrhosis is the prevention of recurrent hepatic encephalopathy (Bass et al., 2010; Bajaj et al., 2021; Caraceni et al., 2021). However, due to the broad-spectrum antibacterial effect (good effect on Gram-positive and Gram-negative aerobic and anaerobic bacteria), many hepatologists believe that rifaximin is a reasonable substitute for quinolones or other systemic antibiotics to prevent spontaneous bacterial peritonitis (Caraceni et al., 2021). Future directions will focus on the treatment of CLDs such as liver cirrhosis with rifaximin, although its impact on the development of HCC is not clear. Some studies have shown that rifaximin can significantly reduce the level of endotoxin, which has a positive effect on NAFLD, cirrhosis and many complications (Kalambokis and Tsianos, 2012; Vlachogiannakos et al., 2013; Gangarapu et al., 2015; Kang et al., 2017). For instance, patients with alcoholic cirrhosis who received rifaximin had a significantly higher 5-year cumulative probability of survival than controls (61% vs. 13.5%, *P* = 0.012) (Vlachogiannakos et al., 2013). At the same time, rifaximin does not seem to increase the antibiotic resistance rate, suggesting the possibility of long-term use (Caraceni et al., 2021; Shamsaddini et al., 2021). In addition, the effects of other antibiotics, such as norfloxacin and isoproterenol, have also been proven. Norfloxacin can prevent spontaneous bacterial peritonitis and reduce mortality in patients with cirrhosis, while the isoproterenol can reduce the expression of carcinogenic gene products by inhibiting STAT3 activation (Dai et al., 2015; Moreau et al., 2018). There are also studies in which the use of bacteriophages to regulate the microbiota for therapeutic purposes is explored. Bacteriophages can target specific bacterial groups while having less interference with symbiotic and nontargeted bacteria (Budynek et al., 2010). At the same time, antibiotic resistance does not convey resistance to phages, and bacteria that are highly resistant to antibiotics can still be effectively inhibited by phages (Albillos et al., 2020). This “tailored” therapy may improve the response to treatment (Helmink et al., 2019).

### Probiotics, Prebiotics and Postbiotics

In the human intestine, probiotics can exert beneficial effects and improve the host microecological balance when given in certain amounts. Lactic acid bacteria (such as species of *Lactobacillus*, *Streptococcus* and *Enterococcus*), *Bifidobacterium*, *Propionibacterium*, *Bacillus*, *Escherichia coli* and *yeast* can be used as probiotics; they can both promote the growth of beneficial bacteria and inhibit the growth of harmful bacteria (Sanders et al., 2010). The beneficial effects of probiotics are often species-specific or even strain-specific (Gibson et al., 2017). Therefore, combining multiple strains in a mixture is the key to therapeutic success. By altering the species and numbers of intestinal flora, probiotics are able to maintain the balance of the gut microbiota, improve intestinal barrier function, and inhibit the occurrence and development of HCC. Studies have shown that probiotic supplementation could be a dietary approach to reduce the occurrence of HCC induced by aflatoxin or diethylnitrosamine in animal models (Zhou et al., 2020). For example, probiotic-fermented milk decreased c-myc, Bcl-2, cyclin D1 and rasp-21 levels and reduced tumour occurrence in rat models (treated with aflatoxin B1 to induce HCC) (Gratz et al., 2006; de Moreno et al., 2007; Zhou et al., 2020).

Probiotics can inhibit the release of proinflammatory factors while increasing anti-inflammatory bacteria and metabolites in the gut. Probiotics inhibit not only the translocation of endotoxins but also the activation of pathogen-associated molecular pattern and damage-associated molecular patterns, such as HMGB1 (Zhang et al., 2012). Much evidence has shown that *Bifidobacterium* is also positively correlated with the high expression of IL-10, playing an important role in liver protection (Cuenca et al., 2014; Liu H.X. et al., 2015). Prohep is a novel probiotic mixture. Feeding Prohep was shown to significantly slow liver cancer growth in mice compared to controls, reducing tumour size and weight by 40%, along with a dramatic decrease in the levels of Th17 cells and the suppression of other angiogenic gene expression in liver tumours (Li et al., 2016). In addition, the antiviral activity of probiotics may reduce the risk of HCC by preventing CHB and CHC (Thilakarathna et al., 2021). Probiotics can upregulate the expression of multiple tumour suppressors and inhibit the expression of oncogenes involved in the pathogenesis of HCC (Thilakarathna et al., 2021).

Prebiotics are foods that selectively stimulate the growth of certain specific microorganisms and have beneficial effects on the host. In mouse models, prebiotics were found to be able to enhance the effects of multiple commonly used chemotherapeutic drugs and radiotherapy (Helmink et al., 2019). Prebiotics also restore microbial community stability and reduce proinflammatory pathways that trigger hepatocarcinogenesis (Zhou et al., 2020).

Postbiotics are defined as a “preparation of inanimate microorganisms and/or their components that confers a health benefit on the host” (Salminen et al., 2021). Studies argue that the beneficial effects of probiotics on health are not necessarily directly related to live bacteria; rather, the bacterial metabolites or bacterial components are promoters of health (Klemashevich et al., 2014). Postbiotics have now been found to protect the gut barrier and suppress intestinal inflammation by modulating multiple mechanisms, such as NLRP6 inflammasome signalling (Tsilingiri et al., 2012; Levy et al., 2015). Perhaps in the future, postbiotics instead of probiotics can be broadly used to improve therapeutic efficacy for HCC.

### Faecal Microbiota Transplantation

Faecal microbiota transplantation consists of the infusion of faeces from a healthy donor to the gastrointestinal tract of a recipient patient to treat a specific disease associated with an alteration in the gut microbiota (Cammarota et al., 2017). FMT has been successfully used to treat *Clostridium difficile* infection (CDI) by mechanisms including direct competition, the stimulation of the mucosal immune system, the restoration of bile acid metabolism, and the repair of the gut barrier (Khoruts and Sadowsky, 2016).

Currently, FMT is being evaluated in trials for various CLDs. A study found that the treatment of mice with alcohol-induced liver lesions with FMT could prevent ALD (Ferrere et al., 2017). Alcohol-sensitive mice have a significant decrease in *Bacteroidetes* in the body following alcohol ingestion, along with an increase in Actinobacteria. Whereas, after FMT, liver damage was alleviated and dysregulated flora was partially restored (Ferrere et al., 2017). FMT (enriched in *Lachnospiraceae* and *Ruminococcaceae*) administration in patients with advanced cirrhosis has been shown to restore antibiotic-associated disruption of microbial diversity and function (Bajaj et al., 2018a). The long-term safety and parameters of cognitive function were consistently improved in patients with cirrhosis treated with post-antibiotic FMT (Bajaj et al., 2019). There were 0 hepatic encephalopathy events in the FMT group compared to 8 in the antibiotic group. Meanwhile, the FMT group exhibited an increase in *Burkholderiaceae* and a decrease in *Acidaminococcaceae* (Bajaj et al., 2019). In addition, some studies have found that FMT can improve the symptoms of NAFLD and play a role in elevating the antitumour immune response (Delaune et al., 2018). However, there are fewer studies on FMT in the treatment of HCC, and it is also unclear whether alterations in the gut microbiota in HCC will be permanently restored by FMT (Zhou et al., 2020). HCC pathogenesis is driven by multiple host factors, greatly complicating the goal of “normalizing” the gut microbiota through FMT (Bajaj and Khoruts, 2020). More animal experiments are therefore needed to demonstrate the efficacy and safety of FMT. A future strategy would be to replace stool for engraftment with an established mixture of bacteria, achieving the same beneficial effect while personalizing microbial manipulation to make it more acceptable to patients and physicians (Bajaj and Khoruts, 2020).

### Immunotherapy

The gut microbiota can dynamically regulate host immunity. Some evidence shows that bacteria in the tumour environment have immune-stimulatory effects, while other studies have shown that bacteria in tumours create an immunosuppressive microenvironment (Li et al., 2019). At present, for some other tumour types, a positive role of the gut microbiota in the antitumour immune response has been found (Zhou et al., 2020). Therefore, the regulation of the gut microbiota may be a new and important auxiliary measure of anti-HCC immunotherapy in the future. Immune checkpoint inhibitors, namely, drugs targeting cytotoxic T-lymphocyte-associated protein 4 (CTLA-4), programmed cell death protein 1 (PD-1) or its ligand PD-L1, are potential therapeutic strategies for HCC (Llovet et al., 2018). A study reported the dynamic characteristics and specificity of the gut microbiota during anti-PD-1 immunotherapy for HCC. Faecal samples from patients responding to immunotherapy showed higher taxa richness and more gene counts than those of nonresponders (Zheng et al., 2019). For dynamic analysis during anti-PD-1 immunotherapy, the dissimilarity of β-diversity became prominent across patients as early as week 6 (Zheng et al., 2019). Therefore, the dynamic changes in the gut microbiota can provide early prediction for the results of immunotherapy for HCC. In addition to the role of the gut microbiota in the immune checkpoint blocking response, there is also an association between the gut microbiota and treatment-related immunotoxicity (Llovet et al., 2018; McQuade et al., 2019). Studies have shown that it is possible to dissociate the impact of the microbiota on therapeutic efficacy from its impact on the adverse effects of CTLA-4 blockers, making it possible to target the microbiota to enhance the effects of CTLA-4 blockade by reducing collateral toxicity (Zhou et al., 2020).

### Precision Microbial Therapy and Its Advantages

The current mainstay of clinical treatment for HCC is surgical resection, chemotherapy, radiotherapy, and interventional therapy. There have also been some studies that have found the influence of the gut microbiota on the therapeutic efficacy of these traditional approaches. For example, the gut microbiota can modulate host responses to chemotherapeutic drugs, and the mechanisms are described as the ‘TIMER’ framework — Translocation, Immunomodulation, Metabolism, Enzymatic degradation, and Reduced diversity (Alexander et al., 2017).

The concept of precision medicine has arisen in contrast to traditional treatment approaches. As the development of precision medicine unfolded, an armamentarium of drugs and an array of molecular targets will set the stage for precision oncology (Prasad et al., 2016). If therapies are guided by a tumour’s genomic alterations, HCC treatment can be markedly improved. The issues of high recurrence, low survival, and poor prognosis with traditional treatment approaches will also be properly addressed. Even then, we end up with the possibility of conquering HCC. Imatinib is arguably the most classical targeted therapy. And now, for HCC, we have this solid direction of the gut microbiota.

Hepatocellular carcinoma pathogenesis is complex, but BAs and immune factors are integral players. The bile acid receptor FXR can negatively regulate BA synthesis and inhibit HCC occurrence. When the intestinal mucosal barrier is disrupted, and normal immune regulation of the microbiota is lost. Large amounts of LPS enter the liver, and HCC is promoted by increasing proliferative and antiapoptotic signals in hepatocytes *via* the TLR4 pathway. Therefore, targeting BAs, LPS and their receptors, which indirectly affect the gut microbiota, seems to be a feasible strategy for the prevention and mitigation of HCC. At present, FXR agonists such as obeticholic acid and TLR4 pathway inhibitors such as eritoran have been proposed to treat liver disease and protect the intestinal mucosal barrier(Kim et al., 2007; Mai et al., 2013; Yu and Schwabe, 2017; Eslam et al., 2019; Younossi et al., 2019; Sun et al., 2021). For instance, 71 (23%) patients given 25 mg obeticholic acid reached the fibrosis improvement endpoint, compared with 37 (12%) in the placebo group (Younossi et al., 2019). Fibroblast growth factor 19 (FGF19) is a downstream signal of FXR, which can activate (FGF receptor 4) FGFR4 and induce hepatocyte proliferation (Sun et al., 2021). Therefore, the development of specific FGFR4 kinase inhibitors, such as BLU-554 (NCT02508467), H3B-6527 (NCT02834780), and FGF401 (NCT02325739), is also a targeted therapeutic approach (Llovet et al., 2018). PI3K/Akt is a TLR4 mediated signal, and targeting its downstream mechanistic target of rapamycin (mTOR) is an emerging direction of research. However, studies have found that the mTOR inhibitor Everolimus does not improve overall survival in patients with advanced HCC (Zhu et al., 2014). This suggests that more in-depth studies are needed to confirm the role of everolimus during HCC development. Taken together, these findings above propose the possibility of using these innovative drugs for HCC.

Other targeted drugs for the treatment of HCC, including sorafenib, regorafenib, lenvatinib and cabozantinib, the anti-angiogenic antibody ramucirumab, and the immune checkpoint inhibitors nivolumab and pembrolizumab, have demonstrated efficacy in clinical trials (Chen et al., 2020). Although these agents bring new hope to HCC patients, their therapeutic effects and treatment options require more in-depth evaluation. An innovative idea is that we can look for the effect between these new drugs and the gut microbiota to connect with the treatment of HCC. Harnessing big ‘omics’ data and artificial intelligence enable profiling of HCC molecular features (such as genome, transcriptome, proteome and metabolome) at different levels, including bulk tissues, animal models and single cells (Chen et al., 2020). We can then use these results to better understand HCC mechanisms, rationally develop new therapies and identify candidate biomarkers of treatment response.

## Conclusion and Expectations

Considerable progress has been made in our studies of the gut microbiota over the past few decades. The gut microbiota widely regulates host physiological functions (participating in bile acid metabolism, regulating immune responses, etc.) and can participate in the development of liver diseases through the gut-liver axis. The occurrence of HCC is currently considered to be the end result of a sustained effect of CLDs. Increasing evidence links CLDs to structural and functional alterations in the gut microbiota, recapitulated by decreases in beneficial species and increases in harmful species.

**FIGURE 4:**
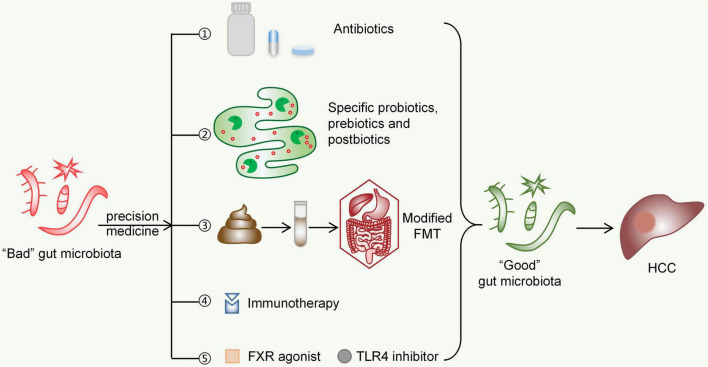
Possible future strategies for targeting the gut microbiota in the treatment of HCC. ➀Antibiotics such as rifaximin, norfloxacin, and others are capable of scavenging specific gut microbes to inhibit HCC progression. ➁Specific probiotics, prebiotics, and postbiotics may inhibit the development of HCC by maintaining the gut microecological environment and improving gut barrier functions. ➂Modified FMT (adjustment of components, delivery frequency, delivery route, etc.) can bring the gut microbiota of HCC patients closer to those of normal people. ➃The immunosuppressive environment shaped by harmful species of the gut microbiota can be improved by immunotherapy targeting CTLA-4 and PD-1. ➄FXR agonists (such as obeticholic acid) and TLR4 inhibitors (such as eritoran) can affect bile acid metabolism and immune response signalling, respectively, both of which might inhibit HCC development by indirectly altering the gut microbiota. FMT, faecal microbiota transplantation; FXR, Farnesoid X receptor; TLR4, Toll-like receptor 4; HCC, hepatocellular carcinoma.

The era of utilizing microbes for diagnosis and therapy is coming. Modulating the gut microbiota is bound to become an important measure for the diagnosis and treatment of HCC in the future. On the basis of the clear associated pathogenesis, we need to better understand the structural and functional changes in the gut microbiota in liver diseases, thoroughly determining their contribution to HCC development. However, it is worth noting that our current understanding of the gut microbiota is largely based on animal models and faecal microbial samples from patients. In addition, the gut microbiota is quite dynamic and affected by a variety of internal and external factors. Therefore, it is necessary to find biomarkers of the gut microbiota in populations of a specific sex or age, from a specific region, and with specific dietary habits. At present, some studies on the characteristic changes in the gut microbiota in HCC have achieved fruitful results (Bajaj et al., 2015b; Ren et al., 2019). In the future, through gene sequencing and machine learning-based big data analysis, we will obtain consistent and reproducible gut microbial signatures, which may serve as HCC biomarkers to assist in early diagnosis (Cammarota et al., 2020). The methods of targeting the gut microbiota for the treatment of HCC, such as probiotics, FMT, and antibiotics, have been described previously. “Precision” drugs targeting HCC are also gradually being developed. Targeting HCC in the future is likely to be a combination treatment modality of traditional approaches plus personalized adjuvant therapy based on the gut microbiota. FMT is currently found to improve the response of multiple cancers to immunotherapy, which paves the way for the treatment of HCC (Riquelme et al., 2019; Baruch et al., 2021). Perhaps the application of modified FMT (adjustment of components, delivery frequency, delivery route, etc.) to intervene in HCC may lead to better outcomes on the basis of safety assurance (Zhang et al., 2018; Figure 4). In conclusion, clinicians can formulate a more optimized treatment regimen based on gut microbial testing for each patient, which also meets the requirements of “personalized medicine.” In clinical practice, the microbiota is modified to regulate gut-liver signals that promote HCC development, ultimately leading to improvements in curative effects and survival for patients.

## Author Contributions

WL drafted the manuscript, performed the selection and organization of literature, and prepared the figures and the table. SG helped to organize references and prepare the table. BW, YZ, JZ, MW, and LS helped to revise the manuscript. BC carried out the design of this review and revised the manuscript. All authors contributed to this manuscript and approved the final manuscript.

## Conflict of Interest

The authors declare that the research was conducted in the absence of any commercial or financial relationships that could be construed as a potential conflict of interest.

## Publisher’s Note

All claims expressed in this article are solely those of the authors and do not necessarily represent those of their affiliated organizations, or those of the publisher, the editors and the reviewers. Any product that may be evaluated in this article, or claim that may be made by its manufacturer, is not guaranteed or endorsed by the publisher.

## Glossary

HCC, hepatocellular carcinoma; CLDs, chronic liver diseases; ALD, alcoholic liver disease; NAFLD, non-alcoholic fatty liver disease; HBV, hepatitis B virus; HCV, hepatitis C virus; CFU, colony-forming units; BAs, bile acids; CA, cholic acid; CDCA, chenodeoxycholic acid; DCA, deoxycholic acid; LCA, lithocholic acid; TRAILR, TNF-related apoptosis inducing ligand receptor; cFLIP, cellular FLICE-like inhibitory protein; mtDNA, mitochondrial DNA; Bcl-2, B cell lymphoma-2; ROS, reactive oxygen species; TLR, Toll-like receptor; ER, endoplasmic reticulum; CaMKII, calcium/calmodulin-dependent protein kinase II; CHOP, C/EBP homologous protein; PKC, protein kinase C; MAPK, mitogen-activated protein kinase; JAK, Janus kinase; STAT3, signal transducer and activator of transcription 3; PLA2, phospholipase A2; AA, arachidonic acid; COX, cyclooxygenase; LOX, lipoxygenase; Egr-1, early growth response gene 1; EGFR, epidermal growth factor receptor; vcm-1, vascular endothelial cell adhesion molecule 1; FXR, Farnesoid X receptor; SIRT1, sirtuin 1; FasL, fas ligand; HNF 1α, hepatocyte nuclear factor 1α; TGR5, Takeda G protein-coupled receptor 5; HSC, hepatic stellate cell; LTA, lipoteichoic acid; SASP, senescence-associated secretory phenotype; DCs, dendritic cells; NKT cell, natural killer T cell; CXCR, C-X-C chemokine receptor type; LSEC, liver sinusoidal endothelial cell;ω-MCA, ω-muricholic acid; NK cell, natural killer cell; LPS, lipopolysaccharide; HMGB1, high-mobility group box 1; MKK4, MAP kinase 4; JNK, c-Jun N-terminal kinase; MMP, matrix metallopeptidase; EMT, epithelial mesenchymal transition; PREX-2, phosphatidylinositol-3, 4, 5-trisphosphate RAC exchanger 2; VEGF, vascular endothelial growth factor; SP1, specificity protein 1; miR-122, microRNA-122; PI3K, phosphoinositide 3-kinase; Akt, protein kinase B; BAMBI, bone morphogenetic protein and activin membrane bound inhibitor; TGF, transforming growth factor; NLRP3, NLR family pyrin domain-containing 3; Th17 cell, T helper 17 cell; Pten, phosphatase and tensin homologue; Treg cell, regulatory T cell; CCL22, C-C class chemokine 22; Sox2, SRY-box containing gene 2; CSC, cancer stem cell; SCFAs, short-chain fatty acids; D_*dys*_, degree of dysbiosis; FMT, faecal microbiota transplantation; CDI, clostridium difficile infection; CTLA-4, cytotoxic T-lymphocyte-associated protein 4; PD-1, programmed cell death protein 1; FGF19, fibroblast growth factor 19; FGFR4, FGF receptor 4; mTOR, mechanistic target of rapamycin.
